# A general framework and practical procedure for improving pxrf measurement accuracy with integrating moisture content and organic matter content parameters

**DOI:** 10.1038/s41598-021-85045-4

**Published:** 2021-03-12

**Authors:** Zengsiche Chen, Ya Xu, Guoyuan Lei, Yuqiang Liu, Jingcai Liu, Guangyuan Yao, Qifei Huang

**Affiliations:** 1 Wuhan University of Science and Technology and Industrial Safety Engineering Technology Research Center of Hubei Province, Wuhan, 430081 China; 2 State Key Laboratory of Environmental Benchmarks and Risk Assessment and Research, Institute of Solid Waste Management, Chinese Research Academy of Environment Sciences, Beijing, 100012 China

**Keywords:** Analytical chemistry, X-ray diffraction

## Abstract

Rapid, accurate detection of heavy-metal content is extremely important for precise risk control and targeted remediation. Herein, a general modeling method and process based on the relationship between Pxrf measured values and site parameters are explored to construct a Pxrf correction model suitable to improve each site’s measurement accuracy. Results show a significant correlation between Pb, Mn, and Zn Pxrf measured values and actual concentrations, with correlation coefficients between 0.8 and 0.93. Through the correlation analysis, the correlation coefficient between the water content and the measured value of pxrf is in the range of 0.2–0.5. Pxrf measurement of all heavy metals was weakly affected by soil organic matter content, with correlation coefficients all lower than 0.5. Model transformation effectively improved the correlation between measured Pxrf value and actual concentration, and transformation increased the correlations of Sr, Mn, and Cu by around 0.11. Model verification results showed that the Pb, Zn, Fe, and Mn models can be used to improve Pxrf method detection accuracy.

## Introduction

In recent years, soil heavy-metal pollution has become one of several global environmental problems. Heavy-metal pollution leads to the deterioration of soil ecosystems, and it is therefore urgent to carry out remediation and risk management^1,2^. The premise of accurate risk control and efficient repair management requires high-precision characterization of pollution^3^. However, high-precision characterization of pollution relies on “massive” and “high-precision” point concentration data^4^. The traditional destructive soil heavy-metal detection method requires complex pre-treatment that is time-consuming, labor-intensive, and costly. These disadvantages have made detection the largest bottleneck restricting the investigation of high-precision pollution sites^5,6^.

The portable X-ray fluorescence spectrometry (Pxrf) method was developed as an attempt to overcome some of the defects of traditional detection methods. It has long been suggested that Pxrf can be used for non-destructive testing^6^. In the Pxrf method, there is no secondary waste in the process of testing, and the cost of testing is far lower than inductively coupled plasma mass spectrometry (ICP-MS), atomic absorption spectroscopy, inductively coupled plasma-optical emission spectrometry, and atomic fluorescence spectrometry ^7–10^. The lower cost makes the use of large-scale soil spatial variability assessment more accurate because more data points can be collected. Pxrf determines element concentrations via the excitation of electrons within the samples by X rays to produce X-ray fluorescence. However, the results of Pxrf detection are easily affected by water content, soil organic matter content, soil particle size, and other factors^6,8–12^. These confounding factors lower detection accuracy^13^.

In view of the existing problems in the Pxrf method, scholars have carried out research from different angles to improve its detection accuracy. By looking for the correlation between the results of Pxrf detection and the results of traditional methods in the laboratory and building a prediction model, it can be used to improve the accuracy of Pxrf detection. The research of Caporale et al.^14^ suggested that there was a good correlation between their linear prediction model and measured metals. Weindorf et al.^15^ showed that Pxrf can be used to predict the salinity of compost. By exploring the specific influence of water content and organic matter on Pxrf detection, Ravansari and Lemke^16^ showed that different kinds of organic matter in soil had different effects on Pxrf measurement. Turner and Taylor^17^ showed that the presence of water can reduce the Pxrf detection signal. Gu et al.^18^ explored the response of spectral characteristics to increases in soil moisture. The Compton Normalization can satisfactorily correct the matrix effect caused by soil water for Zn, As, Rb, Sr, and Pb detection. Studies also show that high-accuracy measurement can be achieved in the Pxrf method by proper sample pre-treatment, standardized preparation, and other instrument calibration^19,20^. In addition to the above point-to-point correction, some scholars have explored some point-to-body studies,that is, using Pxrf to directly improve the spatial interpolation of pollution or improve the estimation of pollution volume. For example, Kim et al.^21^ used a large number of Pxrf detection points as auxiliary variables and improved the accuracy of spatial interpolation of pollutants via the cooperative Kriging interpolation method. Likewise, Zimmer et al.^22^ used X-ray fluorescence spectrometry to determine the spatial distribution of heavy metals in the willow root of contaminated soil.

In general, a large number of Pxrf detection methods have been studied in recent years, and research on measurement accuracy improvement has made some progress. However, the current method for improving Pxrf detection accuracy mainly depends on tedious standard pre-treatment^20^, such as screening, grinding, and drying the soil samples. This pre-treatment inevitably consumes significant amounts of time and precludes the rapid detection of traditional laboratory methods. Therefore, this study explores a general modeling method and process based on the relationship between Pxrf-measured values and site parameters to construct a Pxrf correction model suitable for each site and improve the measurement accuracy of Pxrf. In this paper, the key influencing factors and structural modes affecting the accuracy of Pxrf testing are identified, and stepwise regression is used to construct the relationship model between Pxrf and actual concentrations. The method is applied to the detection of a typical heavy-metal-contaminated site, and the validity and prediction accuracy of the model verified.

## Materials and methods

### Site survey and soil sampling

The research area is a mining site in Xinjiang. Historically, the site was mainly used for the mining of gold and other metals. Owing to the unregulated dumping of tailings, heavy metals became mobilized under the action of rainwater and polluted the soil.

**Figure 1:**
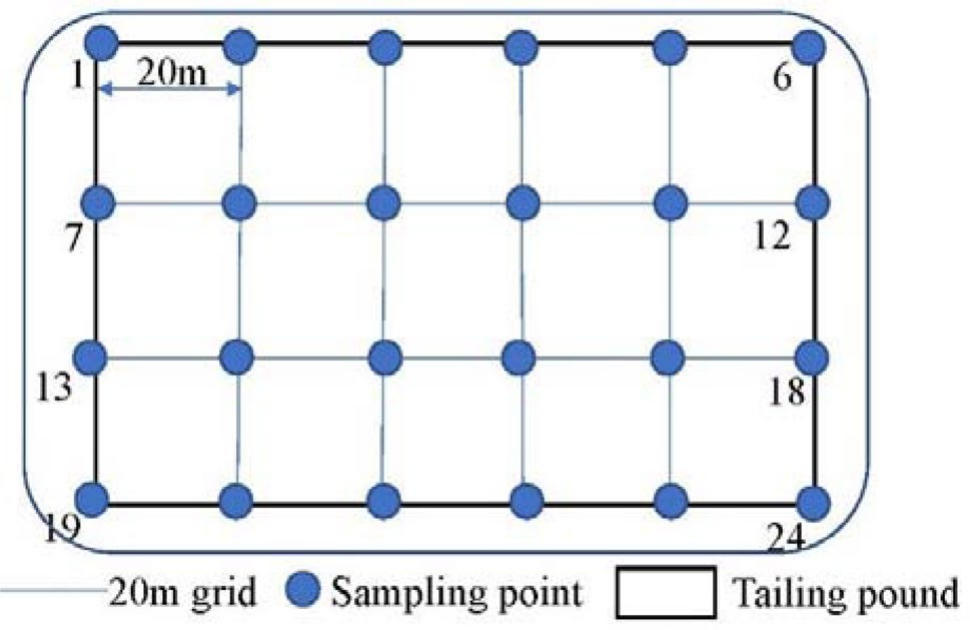
Sampling points.

According to the Chinese standard "The Technical Specification for Soil Environmental Monitoring" (HJ/T 166-2004)^23^, the site layout and sample collection are shown in Fig. 1. Sampling in the horizontal direction was conducted using a checkerboard layout with a spacing of 20 × 20 m. Soil samples were collected of the surface layer (0–0.5 m), shallow layer (1–1.5 m), middle layer (2–2.5 m), and deep layer  (3– 3.5 m). For each layer, 24 samples were obtained (i.e., 96 soil samples in total). Using Pxrf, 96 samples were tested. Among them, 24 samples were used to measure soil moisture content, organic matter, and ICP-MS concentration indices of Pb, Sr, Cu, Fe, Mn, and Zn. Samples of 18 points were selected for modeling, and six random point samples used for verification.

### Framework and practical procedure

The theoretical basis of this method was based on the correlation between the measured results of Pxrf and the actual concentration of pollutants, water content, and organic matter content of soil. Pxrf is affected by many factors and the uncertainty for single factors, and these may exhibit linear or nonlinear relationships (e.g., quadratic, logarithmic, or power). First, the related factors were identified and then the relationship model between Pxrf and these factors built. The model can then be used to correct the detection results of Pxrf and improve its accuracy. It should be pointed out that other factors, such as the content of abundant elements and particle size, will also affect the detection results of Pxrf and the form of the model. Furthermore, there will be different relationship models for different sites. Therefore, the purpose of this paper is not to propose a general model, but rather a general modeling process that can be used to model different sites and different impact variables. This process can then be used to find a relationship model suitable for specific site characteristics to correct Pxrf measurement accuracy.

**Figure 2:**
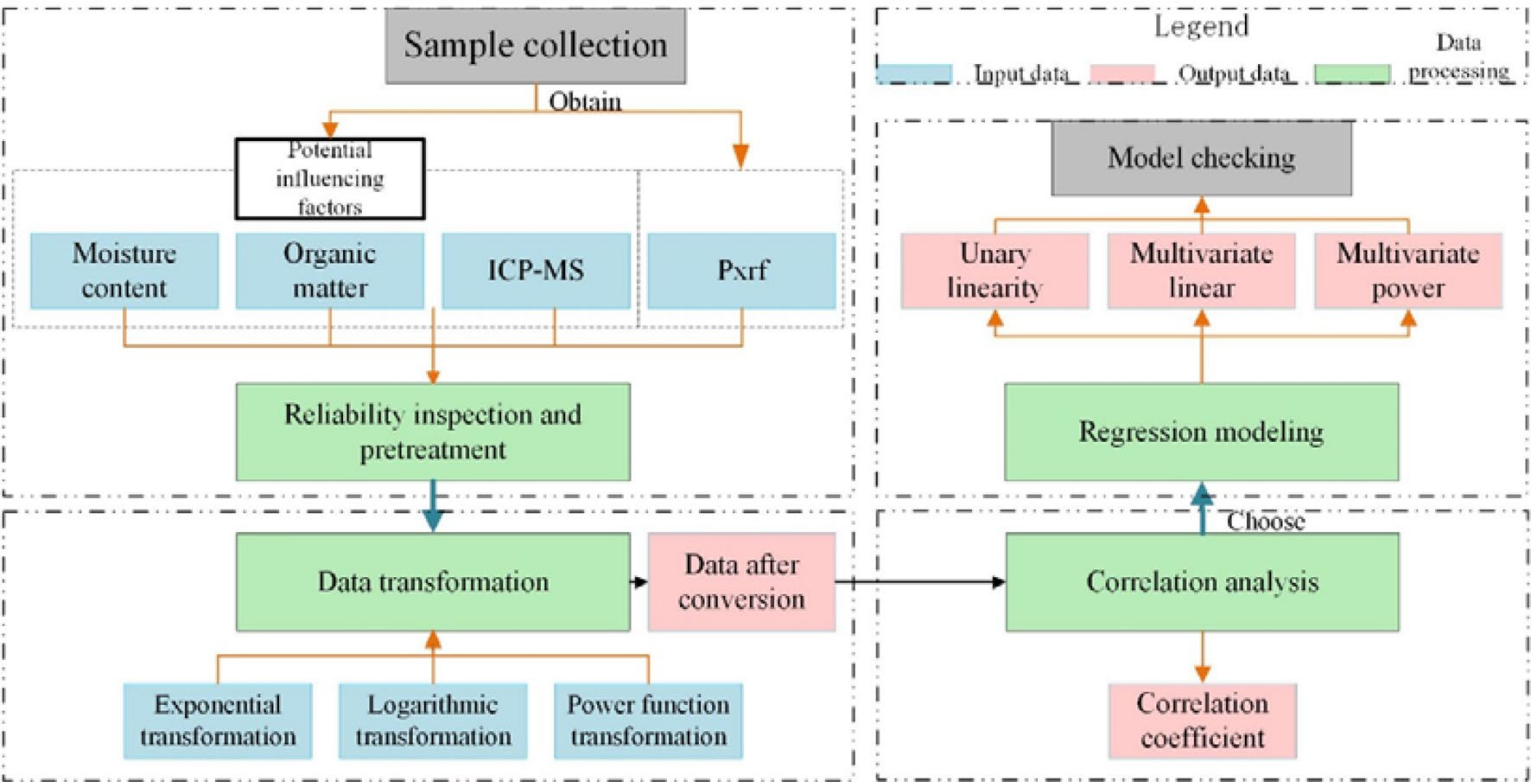
Flowchart of regression model.

The specific research framework and modeling process are shown in Fig. 2, and is detailed as follows.

Step 1: involves selecting samples for modeling and model verification, performing Pxrf detection on all samples, and performing reliability tests and pre-treatment on the analyzed data.
Step 2: involves the mathematical transformation of data points.
Step 3: entails correlation analysis between measurement data and converted Pxrf data.
Step 4: involves selecting the factors with large correlation coefficients to establish different forms of regression models and select the optimal model.
Step 5: involves testing whether the model was significantly correlated with detected metal levels.

### Detection method

A Niton XLT 972 X-ray fluorescence (XRF) spectrometer was used to detect the sample directly. The Pxrf detection mode was the soil mode. The Compton Normalization was used in the Niton PXRF soil mode as a form of internal standardization. Before measurement, we entered the self-inspection page to correct the spectral line drift caused by factors including temperature and aging in the verification process. Self-inspection can be carried out after each startup and preheating to ensure the accuracy of detection. Usually the sample size per scan is 30. In this study, the soil samples at the sub-sampling points were mixed uniformly during the test, and soil samples with a weight of 1 kg were continuously obtained by the quarter method to reduce the variability of the samples. The soil samples were put into polyethylene bags for measurement. The probe window of the instrument was vertically aligned to the center of the sample, and the determination time was set to 30 s. The sample was measured three times and the average value taken in order to consider the influence of human error in actual measurement. The instrument was used to measure at the same location, but the sample was not homogenized. Relative standard deviation (RSD) was used to test the reliability of Pxrf test results^1,24^.

According to the Chinese standard "Solid Waste-Determination of 22 Metal Elements Inductively Coupled Plasma Optical Emission Spectrometry" (HJ781-2016)^25^, microwave digestion was used to digest soil samples and an Agilent 7500a ICP-MS instrument was used to determine the actual concentration of samples. The collected samples needed to be dried, crushed, and passed through a 0.15-mm (100-mesh) nylon screen. We used a 50-mL special digestion tank (with lid) on a low temperature electric heating plate for digestion. The reagents used were: hydrofluoric acid (GR guarantee reagent), concentrated nitric acid (GR), perchloric acid (GR), 1:1 nitric acid solution, and 1% nitric acid solution.

The water content was determined according to the Chinese standard "Method for the Determination of Soil Water Content NY/T 52-1987"^26^, and it is expressed as a percentage. We took a fresh soil sample, mixed it thoroughly, and removed lager impurities such as stones and branches. And then passed through a 0.2-mm nylon screen. The sample was then dried in an oven at 105 °C ± 5 °C to a constant weight.

The organic matter was determined according to the Chinese standard "Method for the Determination of Soil Organic Matter NY/T 1121.6-2006" ^27^(units: g/kg). Under strong acid heating conditions, we used excess K_2_Cr_2_O_2_ standard solution, oxidized the organic carbon in the soil sample, titrated the excess K_2_Cr_2_O_2_ with FeSO_4_ solution. The amount of organic carbon is calculated from the consumed K_2_Cr_2_O_2_ according to the oxidation correction coefficient, and then multiplied by the constant 1.724, which is the content of soil organic matter.

### Data pre-processing

Descriptive statistics of moisture content; organic matter; ICP-MS measurements of Pb, Sr, Cu, Fe, Mn, and Zn; and Pxrf measurements were obtained via SPSS (Statistical Product and Service Solutions)^28^ software (e.g., mean, variance, and kurtosis). Data outlier tests were performed and no outliers were identified. Normal distribution tests (Shapiro–Wilk test) were performed for subsequent modeling.

### Relevance analysis

The correlation analysis included the following aspects, and the correlation analysis used was the Pearson correlation analysis^29^. First, the correlations of Pxrf measurements with soil water content, organic matter, and actual concentrations were analyzed. Second, the correlation analysis of each influencing factor was conducted, and this serves as the basis for screening influencing factors. Finally, logarithmic transformation of Pxrf data; logarithmic and square transformation of ICP-MS data; and logarithmic, square, and cubic transformation of water content and organic matter values were performed and correlation analysis was carried out. Whether the correlation between Pxrf measurements and related factors improved after transformation was observed, and then significant correlation factors were selected to carry out the next step of regression modeling.

### Regression analysis

Regression analysis, based on the correlation analysis^30^, was used to determine the quantitative relationship between Pxrf measurement value, ICP-MS measurement value, and other potential variables. This relationship was then described by establishing a mathematical model. This study is a multiple regression problem, and multivariate nonlinear regression is more difficult. However, the correlation analysis of identified the most relevant model for the relationship between Pxrf and the relevant factors. Therefore, one can directly select the most relevant model for multivariate linear regression modeling.

According to the results of the correlation analysis, different influencing factors can be introduced into the model one by one for regression analysis. The change in model fitting degree (R^2^) after introducing influencing factors can be compared to facilitate the selection of subsequent model types.

## Results and discussion

### Data analysis

The Pxrf measurement values and ICP-MS measurements of six heavy metals (Pb, Sr, Cu, Fe, Mn, and Zn) in soil samples were statistically analyzed. The results are shown in Table 1. The statistical analysis results of water content and organic matter are shown in Table 2. Table 1 shows that the relative standard deviation (RSD) of Pxrf for the determination of heavy metals in soil is in the range 1.2–14.2%, which conforms to "The Technical Specification for Soil Environmental Monitoring" (HJ/T 166–2004) standard. This demonstrates that the method is stable for the determination of heavy metals.

**Table 1:** Statistical characteristics of metal detection in soil.

Statistic	Mn		Pb		Cu		Zn		Sr		Fe	
	XRF	ICP-MS	XRF	ICP-MS	XRF	ICP-MS	XRF	ICP-MS	XRF	ICP-MS	XRF	ICP-MS
MEAN (ppm)	629.431	831.767	51.707	59.600	27.569	30.417	131.222	193.598	141.55	198.12	26.853	40.200
STD. DEV	125.700	134.660	49.996	48.148	6.172	17.291	137.731	231.384	20.02	32.53	4.705	8.941
MIN (ppm)	426.235	615.507	9.684	13.358	16.194	12.985	33.702	54.608	110.00	105.00	19.820	32.000
MAX (ppm)	920.372	1125.214	160.588	148.628	52.000	94.175	435.550	780.649	184.22	230.00	36.610	70.000
Skewness	0.614	0.694	1.331	0.912	2.125	2.583	1.655	1.869	0.00	− 1.456	− 0.232	0.872
Kurtosis	0.341	0.046	0.405	− 0.689	10.136	7.586	1.054	2.117	0.65	2.370	0.475	0.352
Progressive saliency	0.244	0.365	0.509	0.820	0.105	0.054	0.261	0.117	0.822	0.353	0.580	0.838
RSD (%)	4.5–10.8%		2.4–8.7%		2.6–5.9%		1.2–3.5%		5.4–11.2%		7.6–14.2%

**Table 2:** Water content and organic matter statistical characteristics.

Statistic	MEAN	STD. DEV	MIN	MAX	Skewness	Kurtosis	Progressive saliency
Moisture content (%)	3.915	1.898	1.527	8.458	1.026	0.274	0.145
Organic matter (g/kg)	16.063	9.275	3.866	33.368	0.410	− 1.056	0.135

The value of six heavy metals measured by Pxrf was only 47.8–80.3% of the value measured by ICP-MS (Table 1). This may be due to the influence of metal characteristics, soil organic matter, water content, and other factors. The results show that the precision of ICP-MS is ppb level^1^ and the highest precision in Pxrf is ppm level (such as Pb and Cd). Therefore, the ICP-MS value is a quantitative and accurate characterization of the actual concentration.

The normality test of Pxrf and ICP-MS data in SPSS showed that the original data of Pb, Sr, Fe and Mn are normal distribution. The distribution of heavy metals Zn and Cu is also normal after logarithmic transformation.

### Determination of correlation factors and patterns

The correlation analysis between the measured value of Pxrf and the actual concentration as well as its potential influencing factors (moisture content, organic matter) is made, and the results are shown in Table 3.

**Table 3:** Correlation coefficients of Pxrf content of metals and related factors.

Metal category	Mn		Pb		Cu		Zn		Sr		Fe	
Transformation form	Pxrf	ln(Pxrf)	Pxrf	ln(Pxrf)	Pxrf	ln(Pxrf)	Pxrf	ln(Pxrf)	Pxrf	ln(Pxrf)	Pxrf	ln(Pxrf)
1	0.810**	0.797**	0.933**	0.865**	0.214	0.167	0.936**	0.935**	0.376	0.391	0.756**	0.762**
2	0.802**	0.796**	0.954**	0.942**	0.232	0.187	0.986**	0.981**	0.335	0.346	0.756**	0.784**
3	0.811**	0.792**	0.861**	0.772**	0.198	0.148	0.938**	0.877**	0.406	0.425	0.738**	0.726**
4	− 0.471*	− 0.446*	0.194	0.383	− 0.316	− 0.365	0.275	0.317	0.199	0.198	− 0.325	− 0.257
5	− 0.578**	− 0.54**	0.266	0.436	− 0.220	− 0.261	0.356	0.414	0.164	0.163	− 0.365	− 0.297
6	− 0.371	− 0.349	0.109	0.301	− 0.399	− 0.454*	0.150	0.182	0.228	0.226	− 0.283	− 0.216
7	− 0.291	− 0.272	0.034	0.217	− 0.454*	− 0.514*	0.042	0.072	0.250	0.248	− 0.241	− 0.176
8	− 0.083	− 0.039	0.494*	0.629**	0.066	0.047	0.336	0.440	− 0.271	− 0.288	− 0.093	0.010
9	− 0.037	0.000	0.516*	0.671**	− 0.037	0.000	0.391	0.481*	− 0.255	− 0.271	− 0.086	0.020
10	− 0.110	− 0.067	0.441	0.553*	− 0.110	− 0.067	0.250	0.361	− 0.286	− 0.303	− 0.096	0.003
11	− 0.124	− 0.083	0.386	0.479*	− 0.124	− 0.083	0.163	0.276	− 0.297	− 0.316	− 0.096	− 0.002

1 2, and 3 indicate the original value of ICP-MS, logarithmic transformation, and squared transformation, respectively; 4, 5, 6, and 7 represent the original value of water content, logarithmic transformation, square transformation, and cubic transformation, respectively; 8, 9, 10, and 11 represent the original value of organic matter, logarithmic transformation, square transformation, and cubic transformation, respectively; **indicates 0.01 level (two tailed), the correlation was significant; *indicates 0.05 level (two tailed), the correlation was significant.

It can be seen from Table 3 that the original Pxrf measurement values of different heavy metals have a good correlation with the actual concentrations. Among them, the Pxrf values of Mn, Pb, and Zn showed a strong correlation with the actual concentration, and the correlation value was between 0.81 and 0.93. The correlation coefficient of heavy metal Fe was 0.7, showing a moderate correlation. The correlation coefficient of heavy metals Cu and Sr was weak, and the range of correlation coefficient was 0.21−0.376.

In the correlation between Pxrf measurement and water content, the Pxrf measurement of heavy metal Mn has a strong correlation with the water content; the metals Fe and Cu are followed by moderate correlation, and the correlation coefficient is 0.3. The correlation coefficients of heavy metals Sr and Pb are all below 0.2, which is extremely weak correlation.

The correlation between Pxrf measurement of heavy metals Mn, Cu, Fe and organic matter is weakly correlated, and the correlation coefficient is approximately 0.08. The Pxrf measurements of the remaining heavy metals Pb and Zn are moderate correlated with organic matter.

Data transformation has an effect on the correlation between some data, which is manifested in the following three aspects. (1) In terms of the correlation between Pxrf measurement and ICP-MS measurement, the correlation coefficient of heavy metal Zn is increased from 0.936 to 0.986. (2) The correlation between the moisture content of heavy metals Pb Mn Zn and Cu and the detected value of Pxrf is improved after data transformation. (3) The correlation coefficients of organic matter content and Pxrf detection values of Pb, Sr, and Mn increased slightly. Heavy metals Fe and Mn have no significant changes after transformation, and can directly select the original data for modeling. Considering the complexity of the data and the increase of the coefficient, the original data modeling can be appropriately selected.

According to the correlation analysis of each impact factor, the correlation between the various impact factors is weak, so the interaction between the influence factors is not considered in the modeling.

### Establishment of regression model

The original Pxrf measurement value and the transformed value of six heavy metals were used as dependent variables and the influencing factors (actual value, moisture content, and organic matter) were used for regression analysis. The factors with large correlation coefficients were selected, introduced into the model one by one, and the regression model obtained. See Table 4 for the results.

**Table 4:** Solvable coefficients of different metal models.

Model form	Mn	Pb	Cu	Zn	Sr	Fe
Y = f(X)	0.655	0.911	0.054	0.971	0.165	0.571
Y = f(X,b)	0.764	0.915	0.332	0.971	0.186	0.576
Y = f(X,c)	–	0.916	–	–	0.243	–
Y = f(X,b,c)	–	0.932	–	0.972	0.283	–
lnY = f(X)	–	0.887	0.035	0.962	0.181	0.615
lnY = f(X,b)	–	0.901	0.368	0.966	0.200	0.619
lnY = f(X,c)	–	0.952	–	–	0.269	–
lnY = f(X,b,c)	–	0.953	–	0.969	0.307	–

– indicates “not tested,” where y is the concentration of Pxrf, X the concentration of ICP-MS, B the moisture content value, and C the content of organic matter. X, B, and C include the best value after the transformation via logarithm, index, and power function.

From the regression results, it can be seen that the degree of influence on Pxrf detection varied among the modeled factors. From Table 4, it can be seen that the model regression was significant for Pb, Mn, and Zn. The addition of water content as an influencing factor significantly improved the determination coefficient of the Cu regression model, and it also improved the precision of the Mn models. These results are consistent with the results of correlation analysis.

Regression analysis of the adjusted Pxrf measurement shows that the increase in the coefficient of determination of heavy metal Cu was the most significant, and it was 0.27 higher than that before the data adjustment. The influence of data adjustment on heavy metals Zn and Sr is not significant, and adjustment modeling was not considered. In addition, the coefficients of determination for the three heavy metals Pb, Mn, and Fe were slightly increased.

Among the six heavy metals, Cu, Mn, and Sr are significantly affected by water content. Therefore, the water content was included in the model. Organic matter significantly improved the model of Pb, and it is therefore included in the regression model. The regression model of Pb, Fe, and Zn with water content did not significantly improve the accuracy of Pxrf. Therefore, they were not included in the regression model.

Excluding the influence factors that have little effect on the model, the following models can be obtained:

**Table:** 

	Model form
Mn	$\mathrm{X}=\frac{y+94.972\mathrm{ln}b-210.201}{0.648}$
Zn	$\mathrm{X}={e}^{\frac{y+642.479}{160.45}}$
Pb	$X={e}^{\frac{\mathrm{ln}y+0.272-0.449\mathrm{ln}c}{0.767}}$
Cu	$\mathrm{X}={e}^{\frac{\mathrm{ln}y-1.755+0.001{b}^{3}}{0.45}}$
Sr	$X=\sqrt{\frac{\mathrm{ln}y+2.89\times {10}^{-4}{c}^{3}-1.18\times {10}^{-4}{b}^{3}-4.82}{4.059\times {10}^{-6}}}$
Fe	$\mathrm{X}={e}^{\frac{\mathrm{ln}\left(y\right)-0.371}{0.745}}$

### Model test and error analysis

The regression performance indices of the prediction model are shown in Table 5. It can be seen from the table that the regression model coefficients of Pb, Mn, and Zn are significant. However, the regression effect for Sr is poor, and its R value is less than 0.5. The heavy metals Pb, Cu, Zn, Fe and Mn passed the F test, while the heavy metals Sr failed to pass the F test. Therefore, the heavy metals that can be used to establish the model are Pb, Cu, Zn, Fe and Mn.

**Table 5:** Performance indicators of metal regression models.

	Mn	Pb	Cu	Zn	Sr	Fe
R	0.874	0.976	0.607	0.986	0.554	0.784
RMSE	0.6886	0.12	24.689	24.836	112.79	0.2052
Significance F	0.00	0.00	0.02	0.00	0.151	0.01

**Figure 3:**
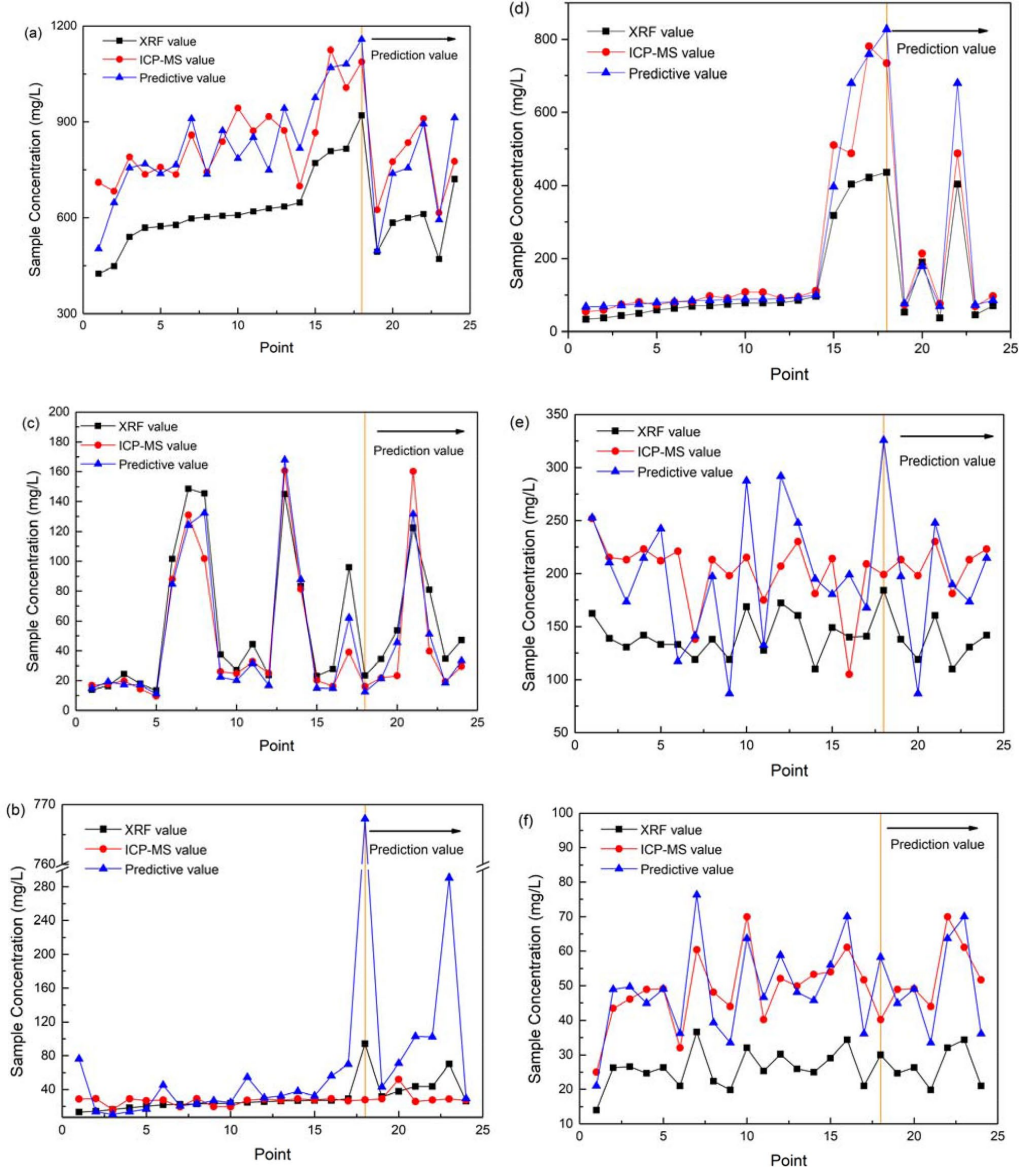
Comparison of model predicted value with original value (**a** Mn, **b** Pb, **c** Cu, **d** Zn, **e** Sr, **f** Fe).

The predicted values of the aforementioned six heavy metals were obtained by substituting the Pxrf measured values from 24 soil samples into the regression model. The calibration and validation sets were entirely independent. A plot of Pxrf measurement value, ICP-MS measurement value, and model prediction value is shown in Fig. 2. The prediction error comparison is shown in Fig. 3, from which it can be seen from that, on the whole, the predicted values of all heavy metals are more accurate than those obtained by the Pxrf measurements. The predicted values from Pb, Mn, and Zn models coincide with the measured values of ICP-MS more closely. The predicted values for Sr and Cu fluctuate more and are less consistent with the ICP-MS values.

**Figure 4:**
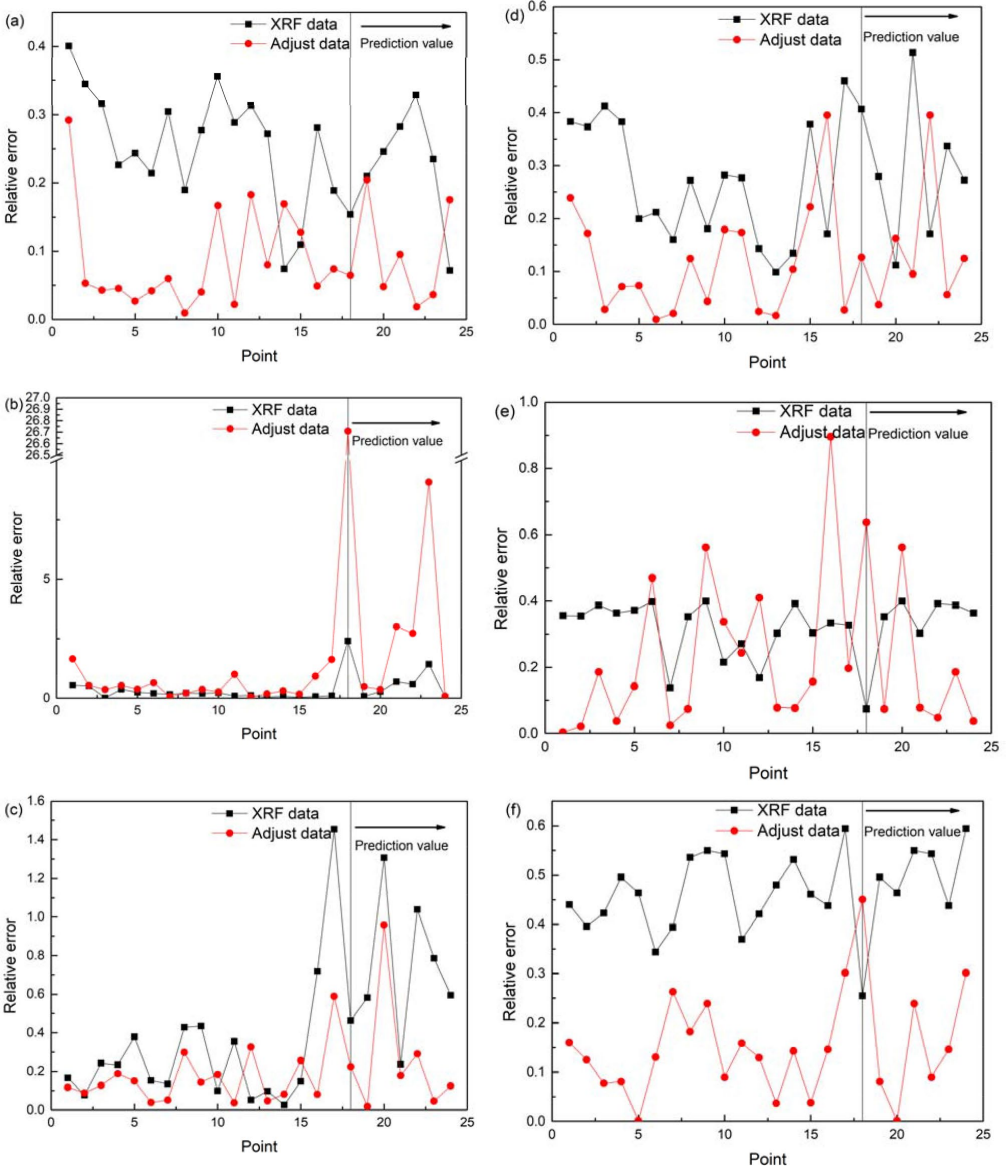
Relative error comparison.

The relative error can better reflect the credibility of measurement. Suppose that the error or absolute error obtained by subtracting the measured true value t from the measured result y is Δ. The relative error can be obtained by dividing the absolute error Δ by the true value t. δ = (Y–t)/tx100% (δ- Relative Error; Y- Measured value; t- True value). It can be seen from Fig. 4 that the relative error between the actual value and the measured value of Pxrf for Sr, Fe, and Cu is greater than 0.4 at most points. After model correction, the relative error of Fe decreased from 0.4 to 0.2, and the relative error of 83% of Zn samples reached approximately 0.1. The heavy metals with larger relative error than the original data is Sr, and this represent poor model fitting.

The average relative errors of six metals are listed in Table 6. It can be seen from Table 6 that the relative errors of Pb and Fe are greatly reduced. The relative error between the predicted ICP-MS data and the actual ICP-MS data is smaller than that between the Pxrf data and the actual ICP-MS data.

**Table 6:** Relative error of different heavy metals.

	Mn	Cu	Pb	Zn	Sr	Fe
Xrf data	0.229	0.517	0.757	0.280	0.366	0.513
Adjust data	0.096	2.62	0.269	0.145	0.164	0.143

It can be seen that models of Pb, Mn, and Zn are good at fitting the data. The data accuracy of Pxrf was significantly improved, and its relative error was reduced 3–8 times. The fit of the model for Fe was not as good, but it could still improve the precision of the model. The models for Sr and Cu exhibited poor performance. The predicted values of these models fluctuate too much compared with the actual value, and they did not consistently produce more accurate values. Therefore, these models are not recommended.

Using actual site data, the appropriate influencing factors were selected and the corresponding mathematical models established. By testing reliability, correlations, and statistical significance, the fitting results of some metals were shown to be reasonable. Pxrf has proved to be a powerful and cost-effective tool for site assessment of metal contamination.

## Conclusions

Through different forms of data transformation processing, the correlation between some metal Pxrf measurement values and the actual values can be improved. After logarithmic transformation, the correlations of the Pxrf measurement values and the actual values increased by 5% for Zn and by 3% for Fe.

After data transformation and selection of influencing factors, four heavy metals (i.e., Pb, Zn, Fe, and Mn) were used to build models. The models for Pb, Zn and Fe are multivariate power equations, while the models for Mn is multivariate linear regression models.

Using the models to adjust values, the error in heavy metal Pxrf data decreased from 22.9 –75.7% to 9.6–26.9%. Because of the particularity of different sites, it is difficult to develop a model that is suitable for every site. Of course, there may be other factors that affect Pxrf detection data. Further in-depth research is still needed to determine additional factors that affect the detection accuracy into the model and to make the model applicable over a wider range of sites.

## References

[1] Congiu, A., Perucchini, S. & Cesti, P. Trace metal contaminants in sediments and soils: comparison between ICP and XRF quantitative determination. (2013).

[2] Zhang X, Sun W, Cen Y, Zhang L, Wang N (2018). Predicting cadmium concentration in soils using laboratory and field reflectance spectroscopy. Sci. Total Environ..

[3] Lemière B (2018). A review of pXRF (field portable X-ray fluorescence) applications for applied geochemistry. J. Geochem. Explor..

[4] Melquiades FL, Appoloni CR (2004). Application of XRF and field portable XRF for environmental analysis. J. Radioanal. Nucl. Chem..

[5] Higueras P (2012). Low-cost geochemical surveys for environmental studies in developing countries: Testing a field portable XRF instrument under quasi-realistic conditions. J. Geochem. Explor..

[6] Shuttleworth EL, Evans MG, Hutchinson SM, Rothwell JJ (2014). Assessment of lead contamination in peatlands using field portable XRF. Water Air Soil Pollut..

[7] Radu T, Diamond D (2009). Comparison of soil pollution concentrations determined using AAS and portable XRF techniques. J. Hazard. Mater..

[8] Bastos RO, Melquiades FL, Biasi GEV (2012). Correction for the effect of soil moisture on in situ XRF analysis using low-energy background. X-Ray Spectrom..

[9] Specht AJ (2018). Feasibility of a portable X-ray fluorescence device for bone lead measurements of condor bones. Sci. Total Environ..

[10] S-M Kim, Y Choi (2017). Assessing statistically significant heavy-metal concentrations in abandoned mine areas via hot spot analysis of portable XRF data. Int. J. Environ. Res. Public Health.

[11] Frahm E (2016). Chemical soil surveys at the Bremer Site (Dakota county, Minnesota, USA): Measuring phosphorous content of sediment by portable XRF and ICP-OES. J. Archaeol. Sci..

[12] Schneider AR, Cancès B, Breton C, Ponthieu M, Marin B (2016). Comparison of field portable XRF and aqua regia/ICPAES soil analysis and evaluation of soil moisture influence on FPXRF results. J. Soils Sediments.

[13] Byers HL, Mchenry LJ, Grundl TJ (2018). XRF Techniques to quantify heavy metals in vegetables at low detection limits. Food Chem. X.

[14] Caporale AG (2018). Monitoring metal pollution in soils using portable-XRF and conventional laboratory-based techniques: Evaluation of the performance and limitations according to metal properties and sources. Sci. Total Environ..

[15] Weindorf DC (2018). Compost salinity assessment via portable X-ray fluorescence (PXRF) spectrometry. Waste Manage..

[16] Ravansari R, Lemke LD (2018). Portable X-ray fluorescence trace metal measurement in organic rich soils: pXRF response as a function of organic matter fraction. Geoderma.

[17] Turner A, Taylor A (2018). On site determination of trace metals in estuarine sediments by field-portable-XRF. Talanta.

[18] Gu R (2019). Impact of soil water on the spectral characteristics and accuracy of energy-dispersive X-ray fluorescence measurement. Anal. Chem..

[19] Rouillon M, Taylor MP (2016). Can field portable X-ray fluorescence (pXRF) produce high quality data for application in environmental contamination research?. Environ. Pollut..

[20] Rouillon M, Taylor MP, Dong C (2017). Reducing risk and increasing confidence of decision making at a lower cost: In-situ pXRF assessment of metal-contaminated sites. Environ. Pollut..

[21] Kim H-R (2019). Better assessment of the distribution of As and Pb in soils in a former smelting area, using ordinary co-kriging and sequential Gaussian co-simulation of portable X-ray fluorescence (PXRF) and ICP-AES data. Geoderma.

[22] Zimmer D (2011). Spatial distribution of arsenic and heavy metals in willow roots from a contaminated floodplain soil measured by X-ray fluorescence spectroscopy. Sci. Total Environ..

[23] China Environmental Monitoring Center & Nanjing Environmental Monitoring Center. Vol. HJ/T 166–2004 38p. A34. The Technical Specification for soil Environmental monitoring (Industry standard - environmental protection, 2004)

[24] Zhou S, Yuan Z, Cheng Q, Zhang Z, Yang J (2018). Rapid in situ Determination of Heavy Metal Concentrations in Polluted Water via Portable XRF: Using Cu and Pb as Example. Environ. Pollut..

[25] HJ781-2016 Solid waste-Determination of 22 Metal Elements Inductively Coupled Plasma Optical Emission Spectrometry. Chinese Standard (2016).

[26] NY/T 52-1987 Method for the Determination of Soil Water Content. Chinese Standard (1987).

[27] NY/T 1121.6-2006 Method for the Determination of Soil Organic Matter. Chinese Agricultural Standard (2006).

[28] The SPSSAU project. SPSSAU. (Version 20.0)[Online Application Software]. Retrieved from https://www.spssau.com. (2020).

[29] Harvey PJ (2018). Delineating the spatial extent of smelter-related atmospheric fallout using a rapid assessment technique. Appl. Geochem..

[30] Radu, T. *et al.* Portable X-ray fluorescence as a rapid technique for surveying elemental distributions in soil. (2013).

